# Contradictory Behavioral Biases Result from the Influence of Past Stimuli on Perception

**DOI:** 10.1371/journal.pcbi.1003948

**Published:** 2014-12-04

**Authors:** Ofri Raviv, Itay Lieder, Yonatan Loewenstein, Merav Ahissar

**Affiliations:** 1 The Edmond & Lily Safra Center for Brain Sciences, Interdisciplinary Center for Neural Computation, Hebrew University, Jerusalem, Israel; 2 Departments of Neurobiology and Cognitive Sciences and the Center for the Study of Rationality, Hebrew University, Jerusalem, Israel; 3 Departments of Psychology and Cognitive Sciences, Hebrew University, Jerusalem, Israel; Oxford University, United Kingdom

## Abstract

Biases such as the preference of a particular response for no obvious reason, are an integral part of psychophysics. Such biases have been reported in the common two-alternative forced choice (2AFC) experiments, where participants are instructed to compare two consecutively presented stimuli. However, the principles underlying these biases are largely unknown and previous studies have typically used ad-hoc explanations to account for them. Here we consider human performance in the 2AFC tone frequency discrimination task, utilizing two standard protocols. In both protocols, each trial contains a reference stimulus. In one (Reference-Lower protocol), the frequency of the reference stimulus is always lower than that of the comparison stimulus, whereas in the other (Reference protocol), the frequency of the reference stimulus is either lower or higher than that of the comparison stimulus. We find substantial interval biases. Namely, participants perform better when the reference is in a specific interval. Surprisingly, the biases in the two experiments are opposite: performance is better when the reference is in the first interval in the Reference protocol, but is better when the reference is second in the Reference-Lower protocol. This inconsistency refutes previous accounts of the interval bias, and is resolved when experiments statistics is considered. Viewing perception as incorporation of sensory input with prior knowledge accumulated during the experiment accounts for the seemingly contradictory biases both qualitatively and quantitatively. The success of this account implies that even simple discriminations reflect a combination of sensory limitations, memory limitations, and the ability to utilize stimuli statistics.

## Author Summary

In this study we explain a previously reported yet unexplained bias in two-alternative discrimination experiments, in which participants are instructed to compare two consecutively presented stimuli. In such experiments, performance is often better when the repeating reference stimulus is in a specific interval, either first or second. However, the "favored" interval differs across studies, depending on seemingly minute details of the experimental protocol. We show that this inconsistency is fully explained as the residual influence of stimuli presented in previous trials, and explain why different experimental protocols yield different results. These findings show that even in the two-alternative procedure, originally developed to eliminate the impact of previous trials, performance is systematically affected by participants' ability to utilize the experiment's statistics of the stimuli, suggesting that sensory processes cannot be studied in isolation, or "out of context": even in the simplest discriminations they involve complex statistical learning that affects participants' performance.

## Introduction

The measurement of perceptual acuity is at the heart of psychophysics. A widely used method for measuring perceptual acuity is the *two-alternative forced-choice* (2AFC) design, in which participants are instructed to compare two sequentially presented stimuli that differ physically along a dimension of interest (e.g., pitch, loudness, duration or brightness). Performance is quantified using a measure known as *Just Noticeable Difference* (JND; also known as Difference Limen, DL, and difference threshold), which denotes the minimal physical difference between two stimuli that is required for attaining a predefined level of performance. Classical Signal Detection Theory applied to psychophysics [1], asserts that if there is no bias in the selection of one interval over the other, then the measured JND in the 2AFC design is independent of internal-criteria and hence is a good method of estimating the limits of the sensory systems. However, a series of studies that are discussed in more detail below have reported several types of bias even when this experimental design is used [2]. Understanding the origins of these biases is important because it can be used as a window for probing the computational principles underlying perceptual processes.

Standard measures of discrimination ability utilize paradigms, in which a constant *reference* (also called *standard*) is presented in one interval and a varying *non-reference* (or *comparison*) is presented in the other interval. While the reported JND is, typically, an aggregate measure that ignores the order of the reference and non-reference stimuli, several studies report that performance level can substantially depend on the temporal interval of the reference stimulus, first or second [3]–[7]. This interval bias has been accounted for as reflecting either better discrimination ability when the reference is in a certain interval, or as a bias favoring one of the possible responses [2], [8] (see also below). However, the direction of preference is inconsistent across studies [2]. Some studies reported better performance in trials, in which the reference is presented in the first interval [3], [8]–[12], whereas others reported better performance when the reference stimulus is presented second ([6], [13], [14] - as re-analyzed by [2]. Note that the presentation of [13], [14] in Fig. 1 of [2] uses the interval of the correct response, and not the position of the reference. Since in [13] subjects had to indicate which interval contained the reference, but in [14] subjects had to indicate which interval contained the non-reference, both experiments actually present better performance in trials in which the reference is second). Therefore, the principles underlying this bias remain elusive.

**Figure 1:**
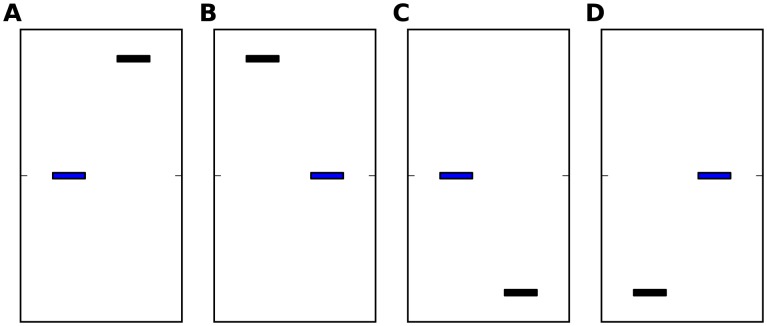
Demonstration of possible stimuli in different 2AFC protocols. Each sub-figure represents a trial type. The blue bar represents the reference stimulus. The black bar represents the non-reference (comparison) stimulus. The left bar in each trial represents the stimulus in the first interval and the right bar represents the stimulus in the second interval. The ordinates denote the magnitude of the stimuli (frequency, in our experiments). **A**: a trial in which the reference stimulus is in the first interval, and the non-reference is higher; **B**: a trial in which the reference stimulus is in the second interval, and the non-reference is higher; **C**: a trial in which the reference stimulus is in the first interval, and the non-reference is lower; **D**: a trial in which the reference stimulus is in the second interval, and the non-reference is lower; In the Reference-Lower protocol only configurations A and B are used, while in the Reference protocol all 4 configurations are equally likely.

Here we hypothesize that the different manifestations of the interval bias can be accounted for in a unified framework of decision making which approximates Bayesian inference, in which noisy sensory signals are integrated with prior expectations [15], [16]. According to this view, the direction and magnitude of the bias reflect the specific history of trials of the experiment. As a result, seemingly benign manipulations of the distribution and order of stimuli used in the experiment have a substantial effect on the perceived magnitude. To test this hypothesis, we conducted two experiments, using two common protocols of auditory two-tone frequency discrimination using the 2AFC design. These two protocols yielded opposite interval biases. However, both results are accounted for by the same decision making model, both qualitatively and quantitatively.

## Results

### Interval bias

We measured frequency discrimination using two 2AFC protocols. In experiment 1 (n = 49) the reference tone (1000 Hz) was always the lower tone (and hence we term it Reference-Lower protocol), resulting in two trial types, depicted in Fig. 1A and 1B. In experiment 2, preformed by a different group of participants (n = 152), this reference tone could be either higher or lower than the non-reference tone (yielding 4 trial types, illustrated in Fig. 1A–1D). We therefore termed it Reference protocol. We denote the frequency of the first tone by 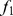 and the frequency of the second tone by 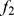. In both experiments we varied the difference between the frequencies of the two stimuli using a 3-down-1-up staircase procedure [17] starting from above-threshold difference, and as expected from this procedure participants answered correctly in approximately 79.4% of the trials in both experiments (80.0% and 80.4% in experiment 1 and experiment 2, respectively). We did not observe any significant differences in the JNDs measured in the two experiments, 4.1%±0.6% and 3.3%±0.5%, in experiment 1 and experiment 2, respectively (mean±SEM across the population; 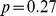, unpaired t-test, 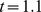, 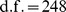).

**Figure 2:**
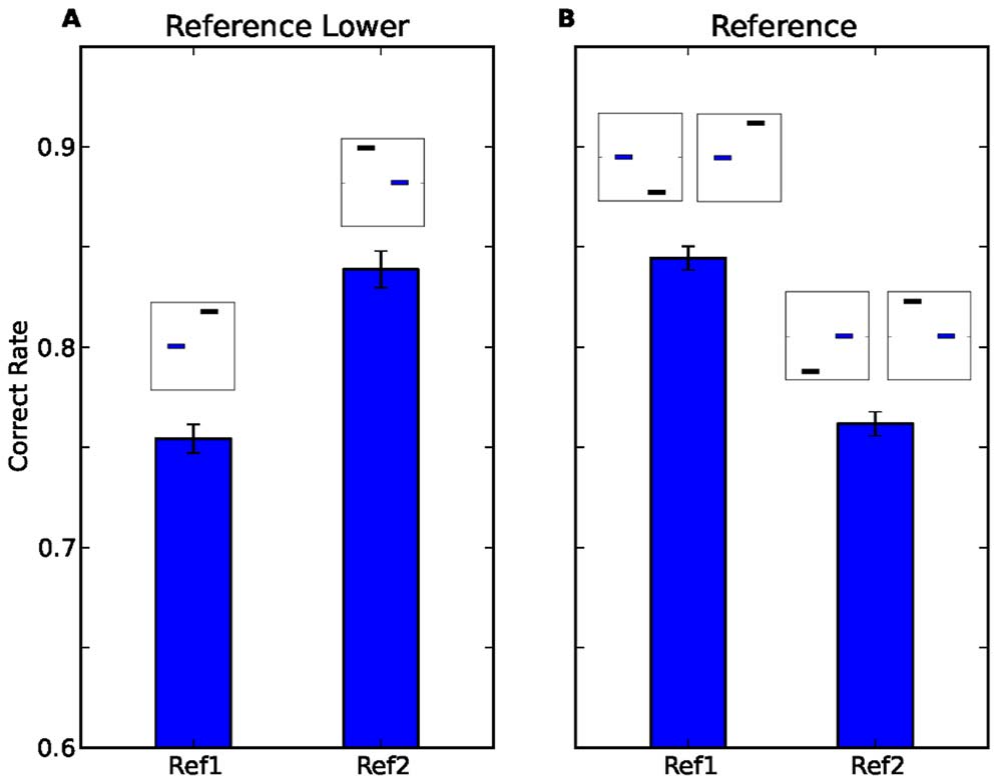
Performance in trials in which the reference is first (Ref1) and trials in which the reference is second (Ref2) in Reference-Lower protocol (A), and Reference protocol (B). Error bars represent the SEM across the participants. The insets above each bar represent the trial types that constitute that bar using the same notation as in Fig. 1. In Reference-Lower protocol performance is significantly better in Ref2 trials, while in Reference protocol it is better in Ref1 trials.

To examine the interval bias in these two protocols, we divided the trials according to the position of the reference, and measured performance in the two groups of trials separately. As presented in Fig. 2A, in the Reference-Lower protocol, when the reference was in the first interval (*Ref1* for short) participants responded correctly in 75.5%±0.7% (mean±SEM across participants) of the trials, compared to 83.9%±0.9% when the reference was in the second interval (*Ref2*; 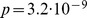, paired t-test, 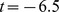, 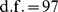). Namely, participants performed better when the reference tone was presented in the second interval. When considering the behaviour of each participant individually, 83% of the participants performed better when the reference was presented in the second interval. However, as shown in Fig. 2B, in the Reference protocol, participants responded correctly more often in Ref1 trials (84.4%±0.6%) compared to Ref2 trials (76.2%±0.6%; 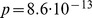, paired t-test, 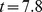, 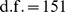). Similarly, 73% of the participants performed better in Ref1 trials. When considering only the trials in the Reference protocol in which the frequency of the reference tone was lower than that of the non-reference (i.e., only the trial types that are also present in the Reference-Lower protocol), the results are similar: participants responded correctly more often when the reference was in the first interval (84.6%±0.8%) compared to when the reference was in the second interval (74.0%±1.1%; 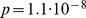, paired t-test, 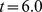, 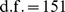). The opposite bias in the two experiments refutes the account of better discrimination ability when the reference is presented second, compared to when it is first [2].

To estimate the magnitude of these biases in the common JND measure, we computed 2 JNDs for each participant, one based only on Ref1 trials, 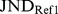, and the other based only on Ref2, 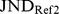, and computed their ratio, 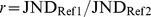. The median of *r* was 0.53 in the Reference protocol (JNDs were lower in Ref1 trials), and 1.27 in the Reference-Lower protocol (JNDs were higher in Ref1 trials).

To ensure that the biases do not stem from the initial, less stable, stages of the assessments, we repeated the analysis presented above, excluding the first 30 trials of each block. By the 30th trial most blocks have already reached a tone difference that is close to the estimated threshold. Still, we found similar results: in the Reference-Lower protocol, participants responded correctly in 72.9%±1.2% when the reference was in the first interval, and in 81.0%±1.2% when the reference was in the second interval (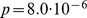, paired t-test, 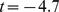, 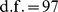). In the Reference protocol participants responded correctly in 80.9%±0.8% when the reference was in the first interval, and in 72.9%±0.8% when the reference was in the second interval (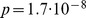, paired t-test, 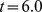, 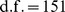). This analysis shows that the biases are not restricted to the initial part of the block, and are relatively stable over time, at least in the time frame of several minutes. They further suggest that the biases are not specific to the experimental design that utilized an adaptive staircase.

**Figure 3:**
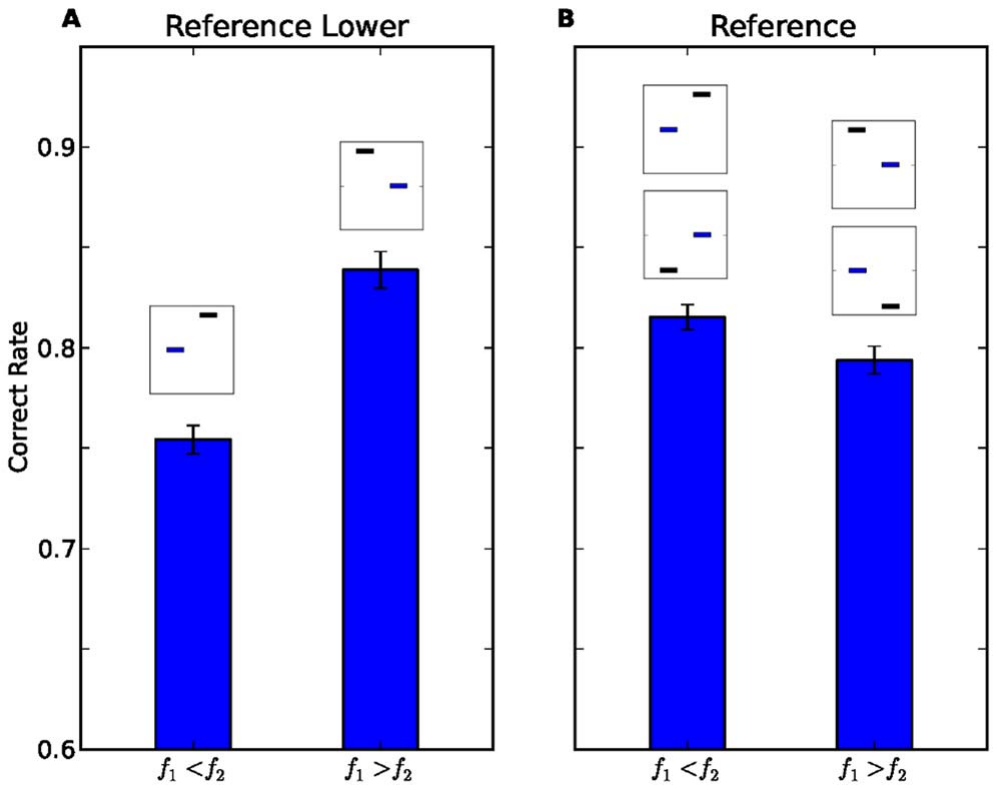
Performance in trials in which first tone is higher than the second (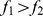) and trials in which the second tone is higher than the first (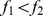) in Reference-Lower protocol (A), and Reference protocol (B). Error bars represent the SEM across the participants. The insets above each bar represent the trial types that constitute that bar using the same notation as in Fig. 1. In Reference-Lower protocol performance is significantly better in 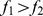 trials, while in Reference protocol it is better in 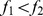 trials.

An alternative account for the differences in performance in the Reference-Lower protocol (Fig. 2A) could have been a bias in favour of responding that the frequency of the first interval is higher (i.e., responding "
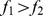
"). The reason is that in the Reference-Lower protocol, Ref2 trials are those in which the correct response is that the frequency of the first tone is higher. If such a bias existed in the Reference protocol, more “
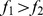
” responses should be observed. This is not the case: participants responded "
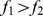
" in 49.2%±0.6% (mean ± SEM across participants) of the trials. To further test whether a response bias exists in the Reference protocol, we separated the trials according to which of the two tones was higher, the first or the second (i.e., the correct response). In the Reference-Lower protocol, this division is equivalent to the division according to the position of the reference (hence Fig. 3A is a re-plot of Fig. 2A). However, in the Reference protocol, the division of the trials according to which stimulus was higher is uncorrelated with the division according to the position of the reference. In contrast to the hypothesis of response bias, participants did not perform better when the first tone was higher. Rather, there was a small, yet significant tendency in the opposite direction: participants responded correctly in 79.4%±0.7% when the first tone was higher, compared to 81.5%±0.6% when the second tone was higher (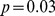, paired t-test; 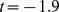, 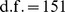, Fig. 3). This result indicates that a response bias, i.e. participants' tendency to respond "
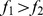
", is not a consistent account of the bias.

Taken together, the differential performance levels when trials are divided according to the position of the reference tone (Fig. 2), and when trials are divided according to the position of the higher tone (Fig. 3) refute the accounts of better perceptual discrimination when the reference is presented first, and of a tendency to respond that the tone presented in the first interval is higher.

### Effect of previous trials on decision and interval bias

In a previous study we demonstrated that the specific history of stimuli presented in the experiment can have a substantial effect on participants' perception in two-tone discrimination tasks [15], manifested as a contraction of memory of the frequency of the first tone in the trial in the direction of preceding stimuli. This bias can be understood in a normative framework as resulting from a Bayesian inference, in which noisy memory of the frequency of the tone in the first interval is combined with the prior distribution of the first stimulus (i.e., the expected stimuli) in order to improve performance. Assuming the prior distribution of stimuli is unimodal, such a strategy is expected to manifest as a contraction of the first tone in the trial towards some memory trace representing the center of the prior distribution of stimuli [16].

**Figure 4:**
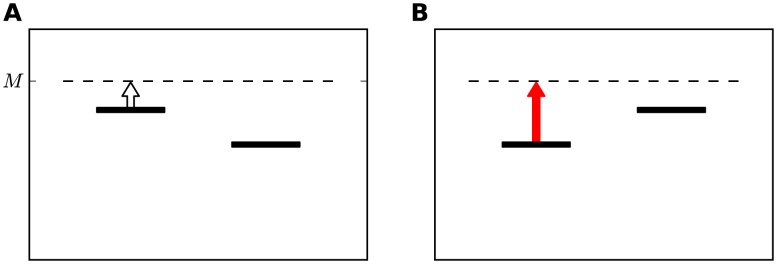
A schematic explanation of the Contraction Bias and its effect on performance. Using the same notation of Fig. 1. The horizontal dashed line represents*M*, the estimated value of the mean stimulus in the block. The vertical arrow presents the contraction of the first stimulus towards the mean value. The arrow is white when this contraction is beneficial to performance (**A**), since it increases the probability of a correct response. The arrow is red when this contraction impairs performance (**B**).

If the memory of the frequency of the first tone contracts towards a prior frequency, the order of the two stimuli within a trial may have a substantial effect on the level of performance. This is illustrated schematically in Fig. 4A and 4B. In this example, the memory trace of previous frequencies (denoted as *M* in Fig. 4) is higher than the frequencies of the two stimuli. This contraction is expected to improve performance when the frequency of the first tone is higher than that of the second tone (Fig. 4A) and to impair performance when the frequency of the first tone is lower than that of the second tone (Fig. 4B). While the Bayesian framework predicts that in these settings, the memory trace, which represents the prior distribution of stimuli should be almost constant, we found, in a different protocol, in which the two frequencies were drawn from a wide distribution and there was no repeated reference frequency, that the frequencies of the first tones in the most recent trials have a substantial effect on the memory trace [15]. These results motivated us to test whether this contraction bias can account for the seemingly conflicting biases presented in Figs. 2 and 3.

**Figure 5:**
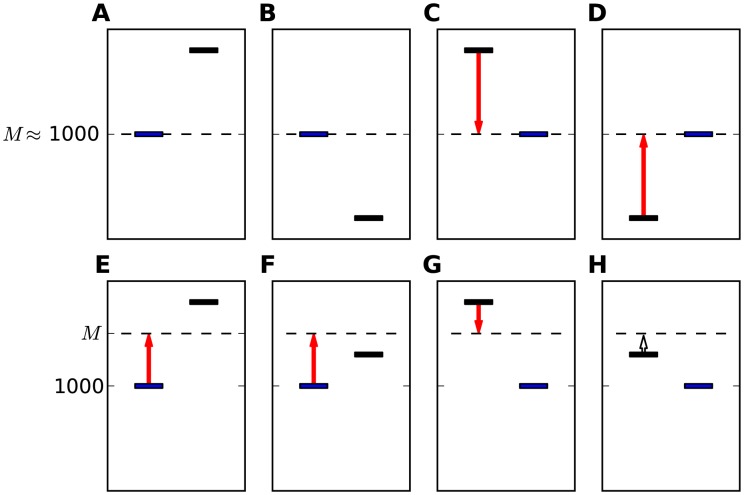
A schematic explanation of the Interval Bias as resulting from the effect of history. Using the same notation of Fig. 1. The horizontal dashed line represents *M*, the memory trace which estimates the value of the mean stimulus in the block. The value of this trace is not the global mean and is expected to vary between trials. The vertical arrow presents the contraction of the first stimulus towards the memory trace. The arrow is red when this contraction impairs performance, since it increases the probability of an incorrect response. The arrow is white when this contraction is beneficial to performance. **A**–**D**: exemplar trials from the Reference protocol; **A**: a Ref1, 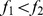 trial; **B**: a Ref1, 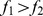 trial; **C**: a Ref2, 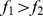 trial; **D**: a Ref2, 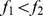 trial; **E**–**H**: exemplar trials from the Reference-Lower protocol; **E**: an easy Ref1 trial; **F**: a difficult Ref1 trial; **G**: an easy Ref2 trial; **H**: a difficult Ref2 trial. Overall, in the Reference-Lower protocol performance is expected to be higher in Ref2 trials, and in the Reference protocol performance is expected to be higher in Ref1 trials.

To see why this could be the case, we commence by considering the simpler case of Reference protocol (Fig. 5A–D). In this case, the comparison tone can be either higher or lower than the reference tone, with probability 0.5. Thus, the memory trace, which corresponds to the weighted average of the frequencies of stimuli in past trials, represents a frequency which is similar to the reference tone. As a result, in Ref1 trials, the contraction is towards the correct value of the stimulus (Fig. 5A,B). By contrast, in Ref2 trials, the contraction of the first tone is towards a value similar to the reference. This will effectively decrease the perceived difference between the tones, which is expected to degrade performance relative to Ref1 trials (Fig. 5C and 5D). This reasoning can qualitatively account for the interval bias of Fig. 2B.

In the Reference-Lower protocol, the comparison tone is always higher than the reference tone. As a result, the memory trace *M*, is expected to be higher than the reference tone. Therefore, the contraction bias is expected to degrade performance in all Ref1 trials (Fig. 5E,F). In Ref2 trials, the situation is more complicated. In easy trials, in which the difference between the two stimuli, 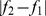 is large, 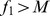 and as a result 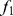 is contracted downwards, towards 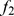, degrading performance (Fig. 5G). By contrast, in difficult trials, in which the difference between the stimuli is small, 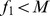, the participants are expected to overestimate the frequency of the first tone, improving performance (Fig. 5H). Overall, better performance is expected in Ref2 trials, compared to Ref1 trials. This analysis is in agreement with the interval bias depicted in Fig. 2A. Taken together, the contraction bias qualitatively accounts for the seemingly contradictory interval biases found in the Reference-Lower and Reference protocols.

### Quantitative account for interval bias

In the previous sections we demonstrated that contraction bias can qualitatively account for the seemingly contradictory interval biases. To test whether it can also account for the interval biases quantitatively, we considered a linear non-linear model, in which the probability of response in each trial is a sigmoidal function of a linear combination of present and past stimuli:
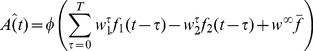
(1)


where 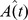 is the probability that the model participant would report that the frequency of the first tone was higher than that of the second tone in trial *t*; *φ* is the normal cumulative distribution function; 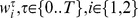 are parameters; 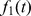 and 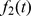 are the logarithms of the frequencies of the first and second tone, respectively, in trial *t*, and 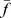 is the mean of the logarithm of frequencies of all stimuli in the experiment until trial *t* (Eq. 1 in [15]).

To better understand this model we note that if 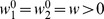 and 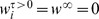, the model is indifferent to the history of the experiment and its choices depend solely on the ratio of the frequencies of the two tones and the internal noise. The parameters 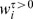 determine the contribution of past stimuli to perception. The larger these parameters are (in absolute value) the larger is the effect of past stimuli in the model. Moreover, the stochasticity of the model depends on the overall values of the weights 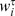. If all weights are equal to zero, 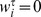 then independent of the stimuli, the probability that the model participant would report that the frequency of the first tone was higher than that of the second tone is 0.5. The larger the weights are (in absolute value) the more deterministic the model is. Stating it differently, scaling the overall weights corresponds to scaling the level of noise in the model.

Naively, in order to test whether the linear non-linear model in Eq. (1) can account for the interval bias, one could fit the parameters of the model for each participant, simulate the resultant model and compare the simulation to the participant behavior. However, because of the large number of parameters 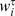 that characterize the model, an agreement between the behaviors of the participants and the simulated model could be interpreted as resulting from over-fitting the parameters of the model. In order to avoid this over-fitting we utilized a different approach. In a previous study, we considered the behavior of 150 participants in a 2AFC discrimination task that was devoid of a reference tone. Using the linear non-linear model (Eq. (1)) with 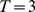, we found the set of weights, denoted as 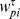 that best accounted for the population behavior (Fig. 3 in [15]). We used the values of the parameters as estimated in [15], in order to constrain the model, and considered a one-parameter model, which we denote as the memory trace model. According to this model,
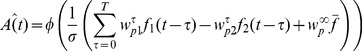
(2)


**Figure 6:**
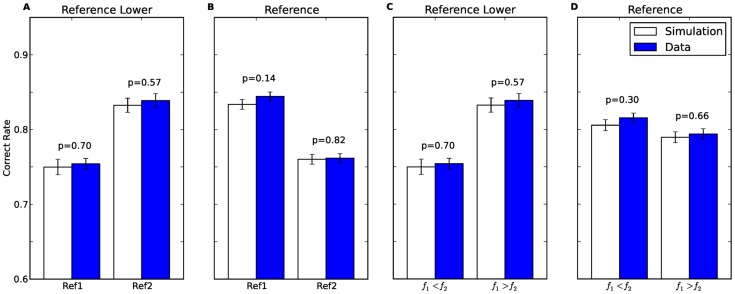
Comparison of model simulations (white) to experimental data (blue). **A**–**B**: Performance in trials in which the reference is first (Ref1) and trials in which the reference is second (Ref2) in Reference-Lower protocol (**A**) and Reference protocol (**B**). **C**–**D**: Performance in trials in which second tone is higher than the first (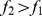) and trials in which the first tone is higher than the second (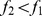) in Reference-Lower protocol (**C**), and Reference protocol (**D**). Note that panel C presents the same data as panel A, due to the equivalence of the Ref1 vs Ref2 and 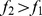 vs. 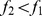 divisions of the trials in the Reference-Lower protocol. Error bars represent the SEM across the participants. p-values are for paired t-tests, uncorrected for multiple comparisons.

Where 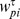 are the parameters estimated in [15], as explained above. The parameter *σ* represents the observer's noise; for each participant, we fitted the model parameter *σ* and simulated the model in the two protocols (for the values of 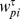 and the details of the fitting, see Methods). Fig. 6 compares the performance of the simulated participants (white) to that of the participants (blue) as a function of the protocol (Reference Lower, A, C; Reference, B, D), divided according to the position of the reference (A,B, experimental data copied from Fig. 2), and the position of the higher tone (C, D, experimental data copied from Fig. 3). Overall, the behavior of the simulated participants was remarkably similar to that of the human participants. To quantify the quality of the fit of the memory trace model to the data, we compared it to a naive model that assumes no history effect:
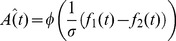
(3)


The memory trace model outperformed the single parameter model in 76% of the blocks in the Reference-Lower protocol (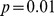, paired t-test, 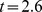, 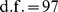), and in 66% of the blocks in the Reference protocol (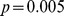, paired t-test, 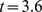, 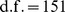). These results substantiate the contribution of the contraction bias to the interval bias.

An alternative account to the interval bias may be a response bias or a shift of the psychometric function. In the framework of a linear non-linear model, this bias can be modelled by adding a constant term *β* to the linear term:
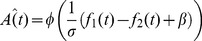
(4)


To test this hypothesis, we fitted the response-bias model (Eq. (4)) to each of the participants and compared the results to the fit of the memory trace model. Because the response-bias model is a two-parameter model whereas the memory trace model is a single-parameter model, we used the Bayesian information criterion (BIC) to compare the two model fits. Figure S1 presents the difference in BIC values between the models as a function of the magnitude of the interval bias in each block. We found that the memory trace model outperformed the response-bias model in 80% of the blocks in the Reference-Lower protocol (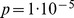, paired t-test, 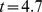, 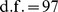), and in 70% of the blocks in the Reference protocol (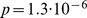, paired t-test, 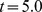, 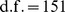), further substantiating our hypothesis that contraction bias underlies the interval bias.

### Response repetition does not account for the observed results

Trials can also be divided into *Repeat* trials, in which the correct response is the same as in the previous trial, and *Alternate* trials, in which the correct response is opposite of that in the previous trial. A possible alternative account of the interval bias could have been a response repetition bias, a tendency to repeat responses. This hypothesis predicts improved performance in Repeat trials compared to Alternate trials. Repeat trials and trials which benefit from the experiment's statistics (Bias+ trials) are highly correlated (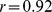) in the Reference-Lower protocol, since a repetition of the correct response means a repetition of the position of the reference. They are also correlated, albeit to a lesser extent, in the Reference protocol (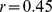). Therefore, in the two protocols presented in this study, it is difficult to distinguish between contraction bias and response repetition.

In order to test the response repetition hypothesis, we re-analyzed previously published results of [15], which used a No-Reference protocol, in which there was no reference stimulus, and both tones are drawn from a wide distribution. In that study, the correlation between Repeat trials and Bias+ trials was substantially lower (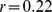) allowing us to distinguish between the two hypotheses. We found that while the difference in performance between Bias+ and Bias- trials was significant and large (88.5%±0.6% vs. 65.4%±0.9%; 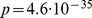, paired t-test, 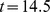, 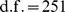) there was no statistically significant difference between performance in Repeat and Alternate trials (79.3%±0.6% vs. 78.5%±0.7%; 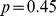, paired t-test, 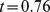, 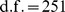). This result supports the hypothesis that it is indeed contraction bias that explains the performance difference, rather than a response repetition effect.

## Discussion

In this study we accounted for long documented yet unresolved behavioral biases in simple discrimination tasks. Individuals performance depends on the position of the constant reference across trials. Yet this dependency seems inconsistent across similar protocols. In the Reference protocol, in which the reference stimulus can be either higher or lower than the non-reference stimulus, performance is better when the reference is presented first, while in the Reference-Lower protocol, in which the reference stimulus is always lower than the non-reference stimulus, performance is better when the reference is presented second. We hypothesized that these biases result from contraction bias. To test this hypothesis, we considered a quantitative contraction model that takes into account the frequency distribution in previous trials and was developed for a different behavioral condition. We found that this model explains the seemingly contradicting directions of the bias without any additional assumptions. According to this model, the change in the direction of the bias stems from the details of the statistics of the stimuli used in each protocol: when the mean of the distribution of stimuli coincides with the value of the reference (as in the Reference protocol), contraction of the first stimulus towards the mean improves performance when the first tone is indeed the reference tone, and degrades performance when the first stimulus is the non-reference; A different asymmetry with respect to the position of the reference is expected when the mean of the distribution of stimuli is different from the value of the reference (as in the Reference-Lower protocol), in line with the observed biases.

### Relation to previous studies

The interval bias in each of the two protocols has been previously reported, separately for each protocol. It has been previously hypothesized that the bias results from response bias or from enhanced perceptual sensitivity when the reference is in a specific interval [2]. However, these explanations cannot account for the opposite directions of the interval bias in the two protocols. We therefore asked whether a model, which we previously derived to account for the effect of recent history on perception in a 2AFC discrimination task that does not use a repeated reference (No-Reference protocol), applies here. In the No-Reference protocol, on each trial, one stimulus is chosen from a uniform distribution between 800 and 1200 Hz, and the other stimulus is controlled by an adaptive staircase, identical to the one used in the current two experiments. We found that rather than performing the discrimination between the stimuli in each trial, as explicitly requested in the instructions and as introspectively reported by participants, listeners incorporated knowledge about the distribution of the stimuli into their perceptual decision. Namely, listeners behaved as if they compared the stimulus presented in the second interval with a linear combination of the frequencies of the first tone in the present trial and the first tone in several preceding trials [15]. This merging of the prior with the representation of the first tone is, in principle, beneficial when internal representations are noisy [16]. We now showed that this concept can also account for the two opposite interval biases in two prevalent protocols. The finding that the same concept quantitatively accounts for performance even when a reference stimulus is introduced suggests that the reference itself does not introduce new mechanisms, aimed for detecting repetition. Recently, a similar model was proposed to account for the interval bias in a "Reference"-like protocol where the non-reference stimuli are symmetrically distributed around the value of the reference (as in our Reference protocol) [8]. It was qualitatively shown that the model predicts the direction of the interval bias in these protocols, where performance is better when the reference is presented first. Using a related, simplified model we now show that the opposite bias, observed in the psychophysically more prevalent Reference-Lower protocol, is also accounted for by the same principle of combining prior knowledge with sensory signals. Based on the same underlying principle as presented here, it can also be predicted that in the Reference-Higher protocol (in which every trial contains a reference, which may be first or second, but is always the higher stimulus) performance should be better in trials in which the reference is second, in line with behavioral findings [6].

### The role of a repeated reference stimulus

The observation that behavioral thresholds are improved when a constant reference is used in the experiment was made more than 70 years ago, and was attributed to the formation of an internal anchor that is based on reference repetition [18], [19]. It led to the almost exclusive usage of reference containing protocols, as means to reveal the "true" sensory bottlenecks. However, several studies have shown that performance depends on the exact trial structure. For example, in another protocol that was not tested in the experiments presented here, called Reference-First protocol, the reference is always presented in the first interval (and the comparison stimulus is either higher or lower). In the Reference-First protocol performance is better than in other reference containing protocols such as Reference and Reference-Lower [8], [10], [11], [20]–[22]. The hypothesis of a formation of an internal reference does not account for this advantage. By contrast, our hypothesis of a simple underlying principle of combining prior knowledge with sensory signals accounts for it, since only in this protocol all trials benefit from this combination. Thus, our simple model accounts for performance both in No-Reference and in different reference containing protocols.

We suggest that the different levels of performance in protocols with and without a reference and the effect of the location of the reference reflect differences in the local history. In the Reference-First protocol and only in this protocol, all trials benefit from this combination. Consequently performance level in that protocol exceeds performance in other protocols. In the No-Reference protocol, in which the distribution of stimuli is broad, it is often the case that the frequencies present in the local history are very different from the current frequency of the first interval, biasing the representation of the frequency of the stimulus in the first interval away from its veridical value. This bias is often detrimental to performance, leading to a substantially higher JND compared with other protocols. Reference and Reference-Lower protocols represent an intermediate situation in which the local history can be either beneficial or disruptive to performance, as we have shown. Consequently, it has only a mild effect on the averaged JND. As a result, the level of performance is worse than that of Reference-First. Compared to the No-Reference protocol, the frequencies in the local history are more narrowly distributed around the veridical value, resulting in a typically smaller difference in performance between trials which benefit (Bias+) and trials which "lose" (Bias-) from experiment's statistics.

### What is a psychometric function?

Results of experiments assessing perceptual resolution are typically reported with the averaging psychometric function (performance as a function of the value of the assessed parameter). When modeling performance in the 2AFC design using a psychometric function it is implicitly assumed that the distribution of stimuli plays no role in perception [23]. By contrast, this work highlights the substantial contribution of protocol design which affects performance through stimuli distribution. The results presented here suggest that sensory processes cannot be studied in isolation, or "out of context", even in simple discriminations measured in isolated laboratory conditions. Whether using a wide distribution of stimuli, or measuring around a reference stimulus, the distribution of stimuli in past trials affects the psychometric curves. Yet, it may be constant with respect to the internal noise and usage of previous trials, as in our model. If that is the case, then given enough data, it should be possible to construct a psychometric function for each of the trial types separately. According to our model, in the Reference protocol, the effect of previous trials should manifest as an effect on the JND (Fig. 5A–5D). In the Reference-Lower protocol, the value of the memory trace is higher than the value of the reference, resulting in both an effect on the JND, and a shift of the point of subjective equality of the psychometric function.

### Putative neuronal mechanism

According to the naive Bayesian model [16], the prior distribution is based on the long history of stimuli presented to the observer. However, our auditory data and our model indicate that the contribution of the past several trials to the memory trace is disproportionately large. Similar results have been reported in the behavior of monkey in a vibrotactile discrimination task [24]. With respect to the contribution of 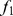 and 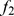 to the formation of the prior, the human auditory experiments and the monkeys vibrotactile experiments seem inconsistent. In the human auditory experiments, behavior is primarily influenced by the frequencies presented as the first stimulus on each trial [15], whereas in the monkey vibrotactile experiments, behavior is primarily influenced by the frequencies presented as the second (most recent) stimulus on each trial [24]. It will be interesting to study whether these differences reflect differences in the sensory modality, specie, or other details of the experiment. When searching for a neural correlate of the prior, we should seek populations of neurons whose activity code the history of the experiment. The signature of a putative prior representing region would be activity whose dependence on the history of the experiment closely follows that of the behavior. In particular, we expect to find neurons that “remember” the history of the experiment over many seconds.

The neural site underlying the integration of the prior, as found in discrimination tasks, remains open. In the auditory pathways, intricate dependencies on experiments history have been observed in neuronal responses as early as the inferior colliculus [25], [26]. Dependencies with time constants of many seconds were amply reported in the primary auditory cortex (e.g. [27], [28]). However, in those experiments the most recent stimulus had the largest effect on subsequent responses, whereas behaviorally we find that the stronger dependence in discrimination tasks is on the history of 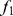 rather than 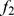. The ability to "bypass" the most recent stimulus when updating the prior may be a property of higher cortical areas. Thus, though this study does not directly map the underlying site, it provides a clear marker for the expected properties of its neural responses.

## Methods

### Ethics statement

The research was approved by the department ethics committee, and all participants signed consent forms.

### Experiment 1: Reference Lower protocol

#### Participants

Data were collected from 49 participants (age 24.3±3.6 years. 17 males) with no hearing problems or learning disabilities. Participants did not have prior experience with the task. Participants were either paid or received course credit for their participation.

#### Experimental design

Perceptual thresholds for auditory 2-tone frequency discrimination were measured using a 2AFC paradigm. On each trial, two 50 ms tones were presented with an inter-stimulus interval of 950 ms. We denote the frequency of the first tone by 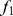 and the frequency of the second tone by 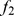. The participants were instructed to report which tone had a higher pitch (frequency) by pressing a corresponding button. Visual feedback of a smiling face or a sad face was presented for 300 ms after correct and incorrect responses, respectively. The subsequent trial began 1 s after the participant's response. Thresholds were assessed in blocks of 80 trials, using an adaptive 3-down 1-up staircase procedure, theoretically converging to 79.4% correct [17]. The initial frequency difference was 20%. The step size (amount of change in % frequency difference between the tones) was decreased every four reversals from 4.5 to 2 to 1 to 0.5 to 0.1%. One of the tones in a trial was a constant reference, set at 1000 Hz. The other tone was determined according to the staircase procedure, and was always higher than 1000 Hz. Namely, the reference could be either first or second (chosen randomly with equal probability, independently between trials), but was always lower than the non-reference. Therefore, the interval containing the reference dictated the correct response - the higher pitch was always presented in the non-reference interval. The two trial types in this protocol (with different positions of the reference) are illustrated in Fig. 1A and 1B. Stimuli were presented binaurally through Sennheiser HD-265 linear headphones using a TDT System III signal generator (Tucker Davis Technologies) controlled by an in-house software in a sound attenuated room in the laboratory. Tone intensity was 65 dB SPL. Discrimination thresholds (Just Noticeable Difference, JND) were calculated as the mean frequency difference in the last 20 trials. Each participant performed 2 blocks of 80 trials (The data presented here is taken from the first two frequency discrimination blocks of experiment 1 of the control group presented in [29]).

### Experiment 2: Reference protocol

#### Participants

Data were collected from 152 individuals (age 22.6 ± 3.8 years. 63 males) that participated in two separate working-memory experiments, conducted at the Hebrew University. Participants did not have prior experience with the task. Participants were paid for their participation.

#### Experimental design

The design of experiment 2 is identical to that of experiment 1, except that the non-reference tone in each trial was chosen randomly to be either higher or lower than the 1000 Hz reference tone. Therefore, this paradigm has 4 trial types, as illustrated in Fig. 1A, 1B, 1C and 1D. The reference could be either in the first or in second interval (as in Reference Lower), and could be the lower (Fig. 1A and 1B) or the higher (Fig. 1C and 1D) stimulus (chosen randomly with equal probability, independently between trials). Thus, the reference interval and the higher interval were not correlated. Each participant performed 1 block of 80 trials.

### Model fitting and simulations

According to the memory trace model, the probability of responding “
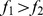
” in trial *t*, is given by 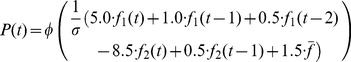



where 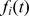 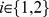 is the logarithm of the frequency of the 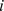-th stimulus presented in trial *t*; *φ* is the standard normal cumulative distribution function; 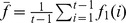 is the mean frequency encountered so far in the block, in the first intervals of each trial; The coefficients of the different terms in the model are based on the experimental results of [15] (obtained in a similar, but not identical, experimental procedure).

The parameter of the model (*σ*) was fitted to each experimental block (80 trials) by maximum likelihood. The minimization was performed using Powell's method, implemented by SciPy [30] version 0.11.0, run from 100 different starting points, normally distributed around (0,0,0) with a standard deviation of 10.

The alternative models considered can be written as: 
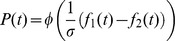
and
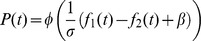
and the fitting of the parameters (*σ* for the first model, and *σ* and *β* for the second model) was done in the same way as described above for the memory trace model.

To generate new datasets of Reference-Lower and Reference protocols, we tested the performance of the fitted models in a Python simulation of our experimental setup, using 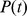 to generate the model's responses, given the frequencies of previous stimuli. Analysis of the resulting simulated data was identical to that of the experimental data. Additionally, Bayesian information criterion value was computed for each of the models for each of the blocks.

## Supporting Information

Figure S1
**Model performance as a function of the magnitude of the interval bias.** Difference in BIC values between the memory trace model and the response-bias model, as a function of the magnitude of the interval bias in each block, for both experimental protocols. In the Reference protocol there was a modest, yet significant correlation (Pearson 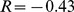, 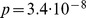, Spearman 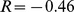, 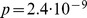). In the Reference-Lower protocol there was no significant correlation (Pearson 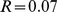, 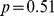, Spearman 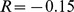, 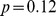). Examining these measures categorically, we find that in the Reference protocol, the memory trace model outperforms the response bias model in 76.6% of the blocks in which the bias is in the expected direction (i.e., higher correct rate in Ref1 trials), and in 52.5% of the blocks in which there was no bias, or bias in the opposite direction (higher correct rate in Ref2 trials). In the Reference-Lower protocol the memory trace model outperformed the response bias model in 81.9% of the blocks in which the bias was in the expected direction (i.e., higher correct rate in Ref2 trials), and in 70.6% in the blocks in which the bias was opposite. Thus, the model has a slight tendency to better account for the behavior of observers that showed the larger bias, or bias in the expected direction compared with observers with smaller bias, or bias in the opposite direction. However, it is evident that the model adequately describes behavior at a single observer level, even for observers that showed no interval bias, or bias in the opposite direction.(EPS)

Dataset S1 **The zip file contains 2 files: reference-lower-data.mat and reference-data.mat containing the data from experiments 1 and 2, respectively.** The files are in MATLAB 5.0 format. Each file contains matrices that are of size Nx80, where N is the number of blocks in that experiment, and 80 is the number of trials in each block. The matrices s1 and s2 contain the values of the first and second stimuli in Hz, respectively, and the matrix c contains 1 for trials in which the participant responded correctly, and 0 otherwise. The file reference-lower-data.mat additionally contains a vector of length 98 with a number identifying the participant that performed each block.
(ZIP)
